# Protocol for *in vitro* co-culture, proliferation, and cell cycle analyses of patient-derived leukemia cells

**DOI:** 10.1016/j.xpro.2024.103202

**Published:** 2024-07-20

**Authors:** Jessica Parker, Sean Hockney, Carly Knill, David McDonald, Andrew Filby, Deepali Pal

**Affiliations:** 1 Department of Applied Sciences, Northumbria University, Newcastle upon Tyne NE1 8ST, UK; 2 Wolfson Childhood Cancer Research Centre, Translational and Clinical Research Institute, Faculty of Medical Sciences, Newcastle University, Herschel Building Level 6, Brewery Lane, Newcastle upon Tyne NE1 7RU, UK; 3 Flow Cytometry Core Facility (FCCF), Newcastle University, Newcastle upon Tyne NE1 7RU, UK; 4 School of Cellular and Molecular Medicine, University of Bristol, Biomedical Sciences Building, University Walk, Bristol BS8 1TD, UK; 5 Biosciences Institute, Innovation, Methodology and Application (IMA) Research Theme, Faculty of Medical Sciences, Newcastle University, Newcastle upon Tyne, UK

**Keywords:** Cell Differentiation, Cell-based Assays, Stem Cells

## Abstract

Leukemia niche impacts quiescence; however, culturing patient-derived samples *ex vivo* is technically challenging. Here, we present a protocol for *in vitro* co-culture of patient-derived xenograft acute lymphoblastic leukemia (PDX-ALL) cells with human mesenchymal stem cells (MSCs). We describe steps for labeling PDX-ALL cells with CellTrace Violet dye to demonstrate MSC-primed PDX-ALL cycling. We then detail procedures to identify MSC-primed G0/quiescent PDX-ALL cells via Hoechst-33342/Pyronin Y live cell cycle analysis.

For complete details on the use and execution of this protocol, please refer to Pal et al.^1^^,^^2^

## Highlights

• Protocol to co-culture patient-derived leukemia cells with human MSCs
• Procedure to track cell proliferation patterns using CTV dye-tagged PDX-ALL
• Steps to identify ALL quiescence using MSC-primed PDX-ALL stained with DNA/RNA dyes

## Abstract (editor highlights)

Publisher’s note: Undertaking any experimental protocol requires adherence to local institutional guidelines for laboratory safety and ethics.

## Graphical abstract

**Figure:**
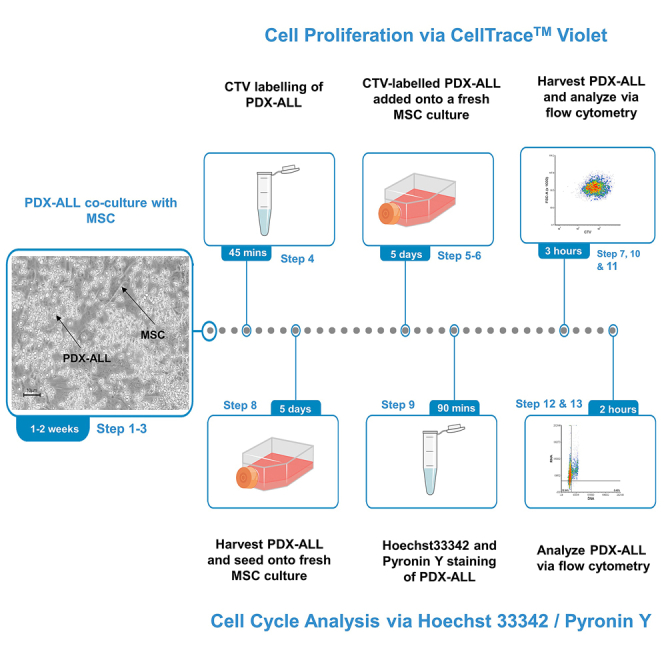

## Before you begin

Here we describe the specific steps used on PDX-ALL cells in co-culture with human MSC (CD105^+^CD90^+^CD73^+^CD45^-^ MSCs, that can differentiate into osteoblasts, adipocytes and chondrocytes), and label PDX-ALL cells *in vitro* with CellTrace Violet, and Hoechst-33342 and Pyronin Y staining to determine cell cycling and quiescence.

This protocol has also been used on PDX-ALL cells, namely samples L707R(relapse) and L707, L49120, MS40, L4967, and ALL cell lines, HAL-01, SEM and PreB697 and AML cell lines, MOLM-14, MV4-11 and Kasumi-1. PDX-ALL samples obtained from mice were confirmed to be positive for hematopoietic marker CD45 and lymphoid marker CD19 using relevant human-specific antibodies. Where relevant a purification using human CD19 antibody was performed using fluorescence-assisted cell sorting (FACS). MSC were isolated as described in previous studies,^3^ from bone marrow of patients undergoing total hip replacements and were confirmed to express CD105, CD90, CD73 and show absence of hematopoietic marker CD45.

This protocol has also been reproduced using ALL and AML co-cultures with different human feeder types including primary human MSC, and induced pluripotent stem cell (iPSC) derived MSC and angiogenic cells. Angiogenic cells refer to a combination of iPSC-derived endothelial and perivascular cells. Co-culture methods used here are as described in previous studies.^1^^,^^3^

### Institutional permissions

The PDX-ALL samples were obtained from the Newcastle Biobank (REC reference number 07/H0906/109 + 5). PDX-ALL samples were obtained from a cryopreserved PDX bank, and samples were generated as per previous projects.^1^^,^^4^^,^^5^ All animal studies to generate PDX-ALL were carried out in accordance with UK Animals (Scientific Procedures) Act, 1986 under project license P74687DB5 following approval of Newcastle University animal ethical review body (AWERB). Obtain appropriate permissions from relevant institutions when conducting similar work.

### Preparation of reagents

**Timing: 15 min**

1. Preparation of CTV Reagents.
  a. Remove a vial containing of CTV Violet from the freezer and centrifuge using brief pulses to ensure that lyophilized pellet constituents did not remain stuck to the cap.
  b. Add 20 μL of DMSO to the CTV, thoroughly mix the contents by vortexing and centrifuge using a brief pulse.

**CRITICAL:** CTV is light-sensitive and must be stored in the dark. All steps containing CTV need to be conducted in the absence of light.

2. Preparation of Hoechst Pyronin Y Reagents.
  a. Dissolve Hoechst 3342 powder in sterile distilled water (dH_2_O) to create a stock concentration of 1 mg/mL. Before each staining protocol, dilute the stock Hoechst 3342 in pre-warmed patient-derived cell media (PDX-ALL media) to obtain a final working Hoechst 33342 concentration of 10 μg/mL.
    **CRITICAL:** Hoechst is light-sensitive and therefore all steps containing Hoechst must be conducted in the absence of light.
    **CRITICAL:** Store 1 mg/mL of Hoechst 33342 stock in −20°C as 1 mL aliquots. Do not repeatedly thaw/re-freeze vials. Store a thawed vial at 4°C and use within 1 week.
  b. Dissolve Pyronin Y in sterile dH_2_0 to create a stock concentration of 0.1 g/mL. Dilute further in pre-warmed patient-derived cell media (PDX-ALL media) to create a final experimental concentration of 100 μg/mL.
    **CRITICAL:** Store stock Pyronin Y in a −20°C freezer in 1 mL aliquots. Store experimental aliquots of 100 μg/mL at 4–8°C, for a maximum of 4 weeks.

## Key resources table

**Table:** 

REAGENT or RESOURCE	SOURCE	IDENTIFIER
**Biological samples**		

PDX-ALL, E2A/HLF	In-house^1^^,^^3^	L707
PDX-ALL, Biphenotypic/MLL rearrangement	In-house^1^^,^^4^	MS40
Human bone marrow mesenchymal stroma	In-house^1^^,^^3^	6255

**Chemicals, peptides, and recombinant proteins**		

Dexamethasone	Sigma-Aldrich, Dorset, UK	D4902

**Software and algorithms**		

FCSExpress Other softwares compatible with .FSC files such as FlowJo	De Novo Software	Link to download: https://denovosoftware.com/full-access/download-landing/

**Other**		

BD FACSCanto II Machine or equivalent flow cytometer such as BD LSR, Cytek Aurora	BD Biosciences	
Low-glucose Dulbecco’s modified Eagle’s medium (DMEM)	Merck, Sigma-Aldrich	D5546
FGF-basic recombinant human protein (10 mg) (bFGF)	BioLegend	571506
Vitronectin XF	STEMCELL Technologies, UK	Catalog #100-0763
Serum-free medium for culture and expansion of hematopoietic cells (SFEM)	STEMCELL Technologies	09655
CellTrace Violet Cell Proliferation Kit	Thermo Fisher Scientific	Cat# C34557
Propidium iodide (PI)	BD Biosciences	51-66211E
Pyronin Y	Merck, Sigma-Aldrich	213519-1G
Bisbenzimide H 33342 trihydrochloride (Hoechst)	Merck, Sigma-Aldrich	Cat# B2261
Trypan blue solution	Gibco	15250-061
Fetal bovine serum (FBS)	Gibco	10500-064
Penicillin-Streptomycin solution	Gibco	15140-122
L-glutamine 200 mM (100×)	Gibco	25030-024
DMSO	Merck, Sigma-Aldrich	D2438-50ML
PBS	Gibco	70011-044
Trypsin-EDTA	Merck, Sigma-Aldrich	15400054
Pluri-strainer mini, 40 μM	PluriSelect	43-10040-60

## Materials and equipment

**Table:** 

MSC medium	Final concentration (v/v)	Amount
Low-Glucose Dulbecco’s Modified Eagle Medium (DMEM)	80%	400 mL
Fetal Bovine Serum	20%	100 mL
Penicillin-Streptomycin	0.1%	5 mL
L-Glutamine	0.1%	5 mL
**Total**	**100%**	**510 mL**

Storage conditions: Prepare Feeder cell medium under sterile conditions using a biosafety cabinet, store at 4°C and pre-warm to 37°C before use. Store at 4°C for up to 1 month.

**Table:** 

Patient-derived cell medium	Final concentration (v/v)	Amount
StemSpan SFEM Medium	99.0%	500 mL
Penicillin-Streptomycin	1.0%	5 mL
**Total**	**100%**	**505 mL**

Storage conditions: Prepare patient-derived cell medium under sterile conditions using a biosafety cabinet, aliquot into 50 mL universal tubes and store at −20°C. Thaw aliquot to 37°C, do not refreeze and store at 4°C for up to 1 month.

**Table:** 

Hoechst solution	Final concentration	Amount
Hoechst 33342	10 μg/mL	100 μL
SFEM Medium	N/A	9.9 mL
**Total**		**10 mL**

Storage conditions: Store at 4°C for 1 week and protect from light.

**Table:** 

Pyronin Y solution	Final concentration	Amount
Pyronin Y	100 μg/mL	1 μL
SFEM Medium	N/A	999 μL
**Total**		**1 mL**

Storage conditions: Store at 4°C for 1 week and protect from light.

**Table:** 

Dexamethasone	Final concentration	Amount
Dexamethasone (1 μM)	10 nM	10 μL
SFEM Medium	N/A	990 μL
**Total**		**1 mL**

Storage conditions: Prepare under sterile conditions using a biosafety cabinet. Store at −20°C. Reconstitute dexamethasone in the compatible medium for the cell type.

**CRITICAL:** Health hazards, may damage fertility or the fetus, powder reconstituted and aliquoted into small amounts to avoid working with powder, PPE to be worn, stored in −20°C freezer and dispose with tissue culture waste.

## Step-by-step method details

### PDX-ALL co-culture with MSC

**Timing: 1–2 week(s) total**

**Timing: 4–7 days for step 1 (dependent on MSC growth)**

**Timing: 2–7 days for step 2 (dependent on PDX-ALL growth)**

**Timing: 15 min for step 3**

1. Culturing and expansion of adherent Mesenchymal Stem Cell (MSCs).
  a. Coat a T25 flask with 10 μg/mL Vitronectin dissolved in 4 mL of MSC media.
    i. Leave the coating on for 12 h at 15–20°C or in the incubator at 37°C.
    ii. Remove excess Vitronectin coating immediately before seeding MSC, being careful not to touch the coated surface, to not disturb the coating.
  b. Remove a cryovial containing MSC from the −80°C freezer and thaw at 15–20°C. Pipette all of the cell contents into a 15 mL universal tube containing 9 mL of fresh MSC media.
  c. Centrifuge the 15 mL universal tube containing MSC at 300 × *g* (1500 RPM) for 5 min.
    i. Aspirate the supernatant and resuspend the MSC cell pellet in 7 mL MSC media freshly reconstituted with 8 ng/mL human FGF-basic (bFGF).
    ii. Transfer this MSC cell suspension to the vitronectin coated T25 flask prepared in 1.a.
      **CRITICAL:** MSC are adherent spindle-shaped cells and adhere to the bottom of Vitronectin coated flasks generally within an hour of seeding.
  d. Change the media in the T25 flask after 24 h, then every 2–3 days, by aspirating old media off and carefully adding fresh bFGF-MSC media.
    i. Continue media changes until MSCs, which are all adherent cells and therefore should stick to the floor of tissue culture ware, reach 80% confluence.
    ii. MSC seeding density of 10,000 cells/cm^2^ yield 80% confluency within 24–48 h.
2. Co-culturing MSCs with PDX-ALL cells to expand PDX-ALL stocks *in vitro.*
  a. Once MSCs are 80% confluent, obtain a cryovial of PDX-ALL cells from the −80°C freezer.
    i. Thaw the PDX-ALL cells at 15–20°C and transfer into a 15 mL universal tube containing 9 mL of pre-warmed patient-derived cell media (reconstituted with 0.1% P/S).
  b. Centrifuge this 15 mL universal tube containing the PDX-ALL cells at 300 × *g* (1500 RPM) for 5 min.
    i. Aspirate the supernatant and resuspend the PDX-ALL pellet in 7 mL of fresh patient-derived cell media at a PDX-ALL seeding density of 1 × 10^6^/mL.
  c. Aspirate the MSC media from the flask containing MSC as prepared in Step 1.d.
    i. Pipette 7 mL of PDX-ALL cells, at a seeding density of 1 × 10^6^/mL, in patient-derived cell media onto the MSC layer.
      ***Note:*** PDX-ALL cells grow in co-cultures as reversible adherent and suspension fractions. Healthy PDX-ALL co-culture appearance includes MSC sticking to the flask as spindle-shaped cells, and rounded PDX-ALL cells attaching to the adherent MSC in a cobble-stone appearance, as described in previous studies.^1^^,^^3^
  d. Count suspension PDX-ALL cells every 48 h, by removing an aliquot of PDX-ALL from the supernatant, followed by a trypan blue exclusion count.
    ***Note:*** Where relevant, add more patient-derived cell media to ensure PDX-ALL-suspension-fraction is maintained at a cellular density of 1 × 10^6^/mL.
3. Harvesting PDX-ALL from MSC co-cultures. We have previously shown that MSC-co-culture primed suspension PDX-ALL have the ability to adhere to MSC when seeded onto fresh MSC feeders and adherent PDX-ALL are able to go back into the media supernatant as PDX-ALL-suspension-fraction cultures.^3^
  a. From PDX-ALL-MSC co-cultures, collect supernatant patient-derived cell media containing PDX-ALL-suspension-fraction cells, into a 15 mL universal tube.
  b. Add 5 mL of 1× PBS to the flask, and wash any loosely adherent PDX-ALL cells. Add these cells to the universal tube described in step 3.a.
  c. Add 0.5 mL of 1× Trypsin-EDTA to the T25 flask and incubate at 37°C, 5% CO2, for a minimum of 2 and up to a maximum of 5 min.
  d. Remove the flask from the incubator and gently tap the sides of the flask to dislodge the MSC and adherent PDX-ALL cells. Collect the dislodged cells into the universal tube described in step 3.a.
  e. Centrifuge the harvested cells at 300 × *g* (1500 RPM) for 5 min and then resuspend pellet in 5 mL fresh pre-warmed patient-derived cell media.
    i. Pass the cells through a PluriStrainer Mini 40 μM cell strainer.
      ***Note:*** This will remove any large MSC clumps and facilitate extraction of a single-cell suspension.
    ii. Purify PDX-ALL cells from this cell suspension via human CD19 antibody staining, a lymphoid cell marker, using either fluorescence-activated cell sorting (FACS) or magnetic-activated cell sorting (MACS).

### CTV labeling of PDX-ALL cells

**Timing: 6 days total**

**Timing: 45 min for step 4**

**Timing: 1 day for step 5**

**Timing: 5 days for step 6**

**Timing: 15 min for step 7**

**Figure 1:**
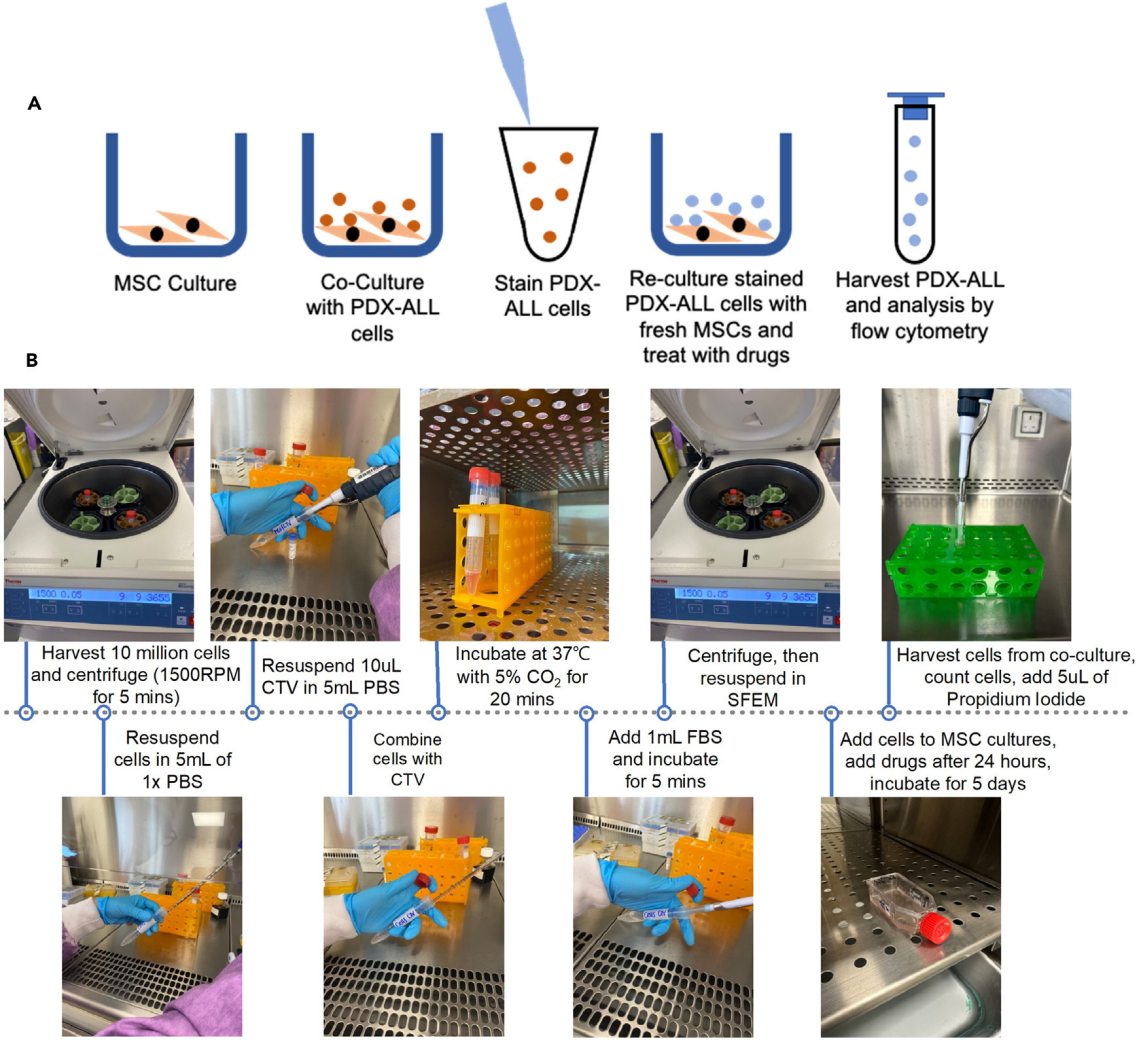
Protocol to stain PDX-ALL with CTV for subsequent MSC co-culture and flow cytometry analysis (A) Schematic detailing the main concept of the CTV staining protocol described within this paper. An MSC co-culture was set-up, which was then co-cultured with PDX-ALL cells. Cells were stained with CTV and re-cultured on fresh MSC. PDX-ALL cells were subsequently treated with compounds as per experimental design. PDX-ALL cells were incubated for the desired time, cells were then harvested and passed through a 40 μM filter before flow cytometry analysis. (B) Images depicting the various steps of the CTV labeling protocol. 10 million PDX-ALL cells were harvested from the MSC-PDX-ALL co-culture. Cells were centrifuged at 1500 RPM for 5 min. PDX-ALL cells were resuspended in 5 mL of 1× PBS and separately, resuspend 10 μL of CTV in 5 mL of 1× PBS. The PDX-ALL cells were combined with CTV solution, then incubated for 20 min. 1 mL of FBS was added before cells were incubated for 5 min. CTV-labeled cells were centrifuged and then resuspended in 20 mL of fresh SFEM media. The CTV-labeled PDX-ALL cells were then divided equally between experimental arms, drugs were added (if required) and then incubated for 5 days. PDX-ALL cells were harvested, filtered and 5 μL of propidium iodide was added before flow cytometry analysis.

This section details the labeling of PDX-ALL cells, sample MS40, with CTV, the experimental set up and harvesting of the PDX-ALL from MSC-ALL co-cultures. The CTV labeling protocol is referred to in Figure 1.

4. CTV labeling of PDX-ALL cells.
  a. Perform trypan blue exclusion counts on MSC co-culture primed and human CD19-purified PDX-ALL cells.
  b. Pass 10 million CD19^+^ PDX-ALL cells through a 20 μM pluri-strainer filter.
  c. Centrifuge the PDX-ALL cells at 300 × *g* (1500 RPM) for 5 min. Aspirate the supernatant and resuspend the cell pellet in 5 mL of 1× PBS.
    **CRITICAL:** The number of cells and volumes of reagents will differ depending on experimental set-up, adjust reagent volumes within ratio as required.
  d. In a fresh universal tube, add 10 μL of CTV to 5 mL of PBS.
  e. Add the 5 mL CTV/PBS solution to the 5 mL PDX-ALL cell suspension from step 1.c., and incubate for 20 min at 37°C and 5% CO_2_.
  f. Add 1 mL FBS to the CTV-stained PDX-ALL and incubate at 37°C, 5% CO2 for 5 min.
  g. Centrifuged the cells at 300 × *g* (1500 RPM) for 5 min and remove the supernatant.
  h. Resuspend the CTV-stained PDX-ALL cells in fresh patient-derived cell media to produce a PDX-ALL seeding density of 0.5 × 10^6^/mL, in a final volume of 20 mL patient-derived cell media.
  i. Acquire a small aliquot of CTV-stained PDX-ALL for analysis by flow cytometry (see step 7) to assess presence of start-point CTV dye intensity.
5. Co-culture of CTV labeled ALL cells with MSC.
  a. Remove MSC media from two T25 flasks of freshly cultured 80% confluent MSC.
  b. Acquire the CTV labeled PDX-ALL cells from step 1.h. Divide these cells equally between the experimental arms, control and dexamethasone treatment.
    i. Carefully seed these CTV-stained PDX-ALL, in 10 mL patient-derived cell media, at a PDX-ALL seeding density of 0.5 × 10^6^/mL, on top of the MSC-layer in each T25 flask.
  c. Incubate at 37°C with 5% CO_2_ for 24 h.
6. Addition of drugs to co-culture.
  a. 24 h following the completion of step 2., aspirate all of the 10 mL of the supernatant patient-derived cell media containing all of the PDX-ALL-suspension-fraction cells from the T25-MSC-co-culture flask, into a 15 mL universal tube.
  b. Centrifuge the cells at 300 × *g* (1500 RPM) X 5 min.
    i. Resuspend the cells in 10 mL of fresh patient-derived cell media containing 10 nM dexamethasone.
  c. Immediately pipette this dexamethasone-containing patient-derived cell media along with the suspension
    i. PDX-ALL cells, back into their respective T25-flasks containing the MSC and MSC-adherent PDX-ALL cells.
  d. Incubate at 37°C with 5% CO_2_ for 5 days.
7. Isolation and harvesting for analysis.
  a. Harvest 1 × 10^6^ PDX-ALL cells from each experimental arm, as described in “PDX-ALL co-culture with MSC”, step 3.
    ***Note:*** Flow cytometry analysis only needs 1 mL of cell suspension at a density 0.25 × 10^6^/ml, therefore cells may need to be centrifuged, media aspirated and resuspended in appropriate volume of media as per harvest cell counts.
  b. Pass the CTV-tagged and MSC-co-culture primed harvested PDX-ALL cells through a 40 μM pluri-strainer into a standard 5 mL FACS tube and add 5 μL of Propidium Iodide.
    **CRITICAL:** Addition of propidium iodide is essential to gate out dead cells.
  c. Analyze via flow cytometry using the BD Biosciences FACS Canto II machine. Other alternative equipment compatible with this protocol would include BD LSR, Cytek Aurora.

### Hoechst Pyronin Y staining of PDX-ALL cells

**Timing: 7 days total**

**Timing: 7 days for step 8**

**Timing: 90 min for step 9**

**Figure 2:**
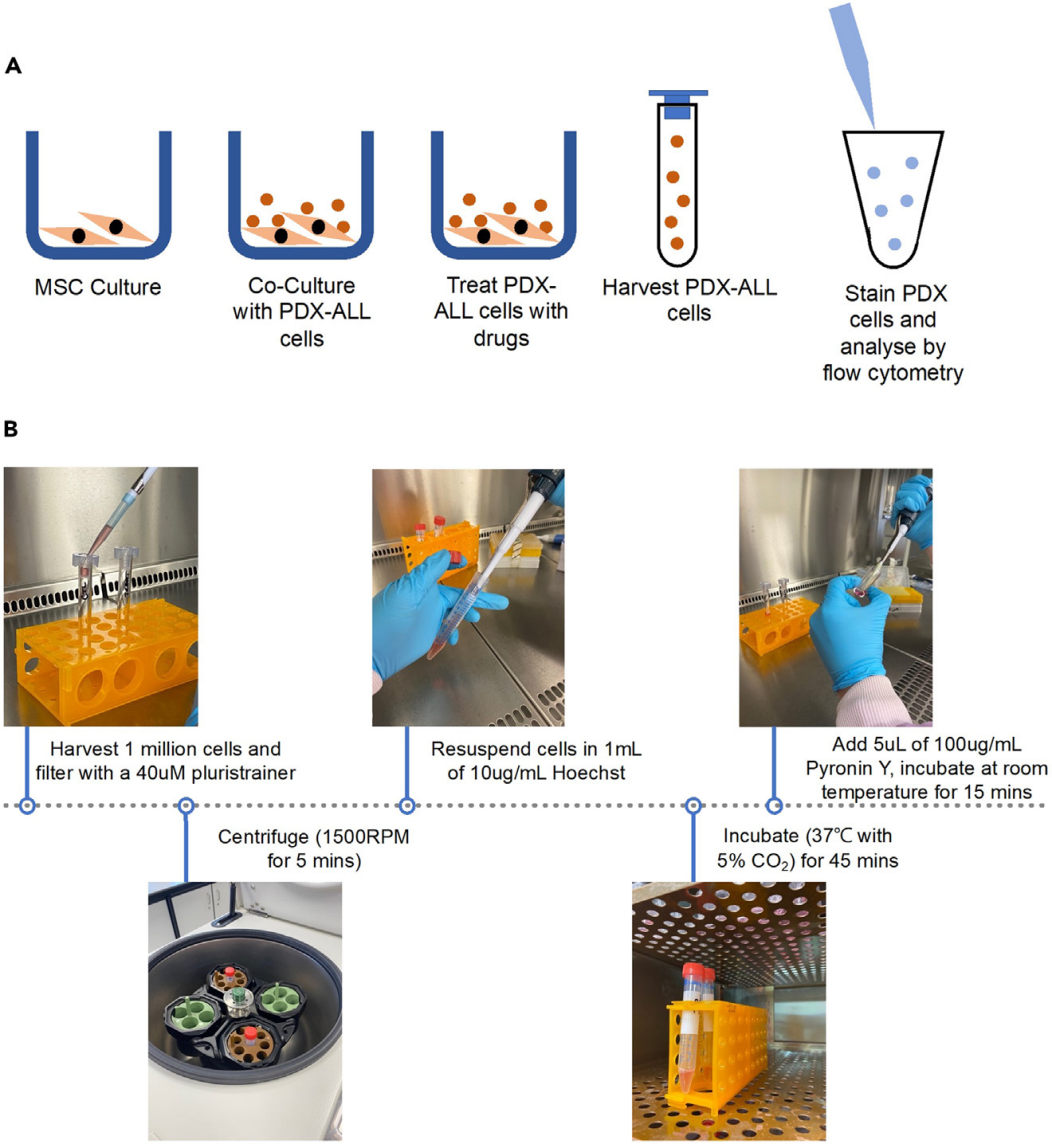
Schematic detailing the main concept of the Hoechst Pyronin Y staining protocol described within this paper (A) MSC culture was set up and then co-cultured with PDX-ALL cells. PDX-ALL cells were then treated with drugs (if required) and incubated for the desired time. Cells were then harvested, filtered and stained, before flow cytometry analysis. (B) images depicting various steps of the Hoechst Pyronin Y staining protocol. MSC-PDX-ALL co-cultures were set up and incubated for five days. 1 million cells were harvested from co-culture and passed through a 40 μM filter. PDX-ALL cells were centrifuged and resuspended in 1 mL of 10 μg/mL Hoechst. PDX-ALL cells were then incubated for 45 min. 5 μL of 100 μg/mL Pyronin Y was the added to Hoechst stained PDX-ALL cells before flow cytometry analysis.

This section details the experimental set-up, harvesting and staining of PDX-ALL cells, L707. Hoechst Pyronin Y staining is an end-point experiment; therefore co-cultures were initially set up for each experimental arm, control and dexamethasone treated, and staining occurred at harvesting after 5 days. However, this process of cell cycle staining does not involve cell fractionation and therefore viable PDX-ALL cells can be flow cytometry sorted for subsequent live cell fate tracking or analysis. The Hoechst Pyronin Y staining protocol is referred to in Figure 2.

8. Co-culture set-up.
  a. Count PDX-ALL using steps from section “PDX-ALL co-culture with MSC”, step 2.d.
  b. Set up control and dexamethasone-treated MSC-PDX-ALL experimental arms as described in previous sections. Maintain PDX-ALL seeding cell density at 0.5 × 10^6^/mL.
  c. Incubate untreated control and 10 nM dexamethasone treated arms at 37°C with 5% CO_2_ for 5 days.
9. Hoechst staining of MSC co-culture primed PDX-ALL cells.
  a. Harvest 1 × 10^6^ PDX-ALL cells from the co-culture. Filter the cells through a 40 μM pluri-strainer and centrifuged at 300 × *g* (1500 RPM) X 5 min.
  b. Resuspend the MSC-co-culture-primed PDX-ALL cell pellet in 1 mL patient-derived cell media containing 10 μg/mL Hoechst 33342 and incubate at 37°C with 5% CO_2_ for 45 min.
  c. Add 5 μL of 100 μg/mL Pyronin Y directly to the Hoechst 33342 stained cells and pipette into a standard 5 mL FACS tube.
  d. Store stained cells on ice and protect from light, before analyzing through flow cytometry using the BD Biosciences FACS Canto II machine. Other alternative equipment compatible with this protocol would include BD LSR, Cytek Aurora.

### Proliferation analysis of CTV labeled PDX-ALL cells

**Timing: 2 h total**

**Timing: 1 h for step 10**

**Timing: 1 h for step 11**

**Figure 3:**
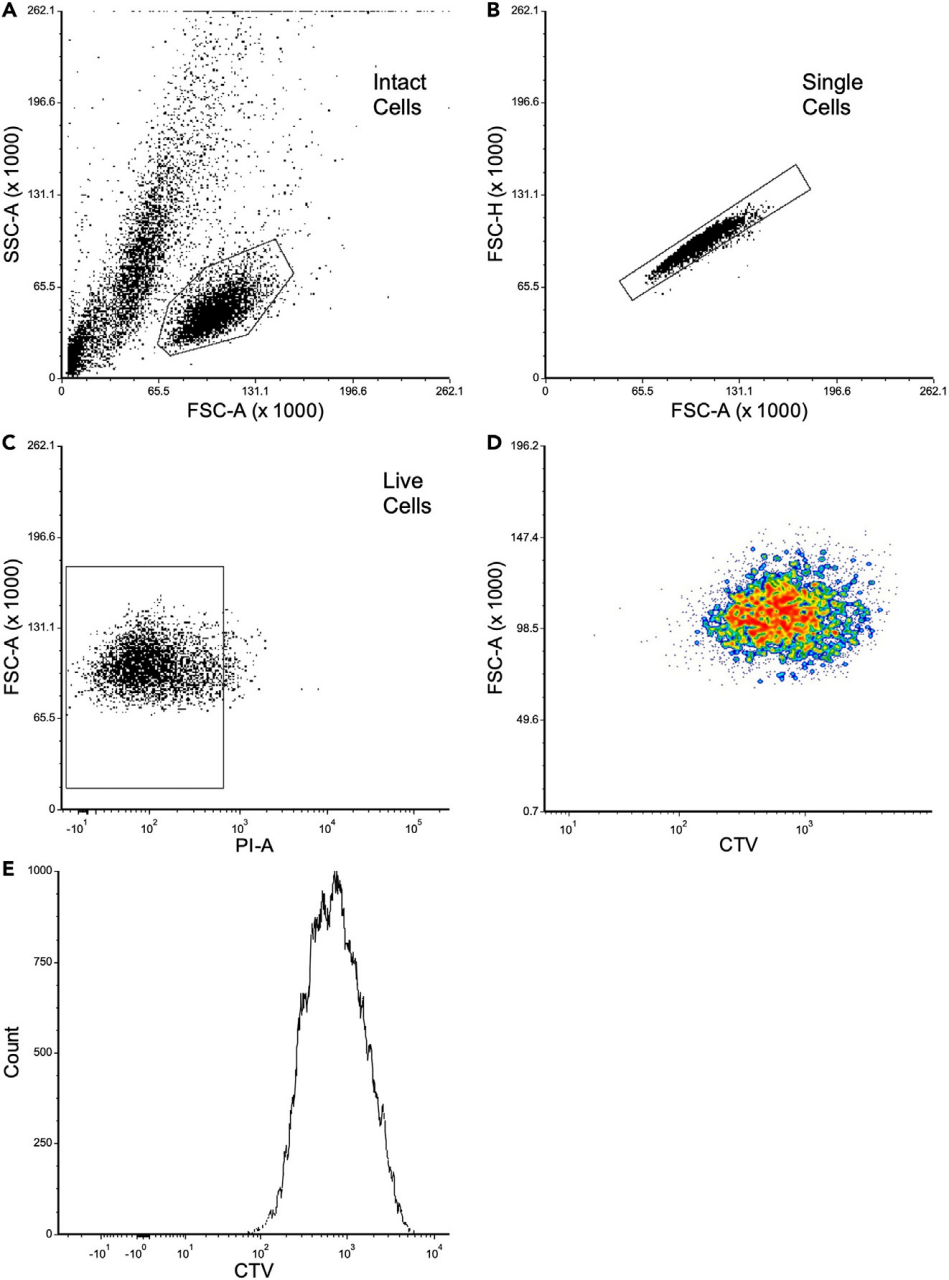
FCS Express analysis of CTV labeled PDX-ALL cells (A) SSC-A versus FSC-A to gate intact cells. (B) FSC-H versus FSC-A to gate single cells. (C) FSC-A versus PI-A to gate live cells. (D) FSC-A versus BV421-A to show proliferation with the cells to the far right indicating a retention of CTV labeling. (E) A histogram used to show cell proliferation. **Note**: The results from this protocol were analyzed using FCS Express 7 software, however this can be done in any flow cytometry analysis software, the principle of each step will be the same.

This section details the proliferation analysis of the CTV labeled PDX-ALL through flow cytometry. This section refers to Figure 3.

10. Flow Cytometry analysis using the FACS Canto II machine. Other alternative equipment compatible with this protocol would include BD LSR, Cytek Aurora.
  a. Set-up the FACS Canto II machine using the standard SOP from BD Biosciences.
  b. From the browser toolbar, select the “new experiment” toolbar (or access a premade experiment template).
  c. Select “New Specimen” icon to view the tube, select the arrow next to the relevant sample tube, which should turn green and is now active.
  d. Tabs will now appear in the Cytometer window, select the Parameters tab.
    i. As a default Forward scatter (FSC)/side scatter (SSC) are in linear scale and fluorescence is in log scale.
  e. Use the worksheet window to create dotplot and histogram templates by clicking the icon and then clicking on the global worksheet, the X or Y axis by right clicking on the name of the axis. Create gates using the menu bar.
  f. Resuspend cells within the control sample tube, and load the tube onto the sample injection tube (SIT), and use the acquisition dashboard to ensure the flow rate is low, SIT flush is selected, number of events is 10,000 and then set to ‘acquire data’.
  g. Adjust the FSC and SSC voltages in the ‘instrument’ window as required so the intact cells are gated/selected for data capture and analysis (Figure 3A).
  h. Use suspension ALL cells to determine the FSC/SSC parameters to specifically gate ALL cells for subsequent analysis.
    i. Use the violet laser (405 nm) to measure the intensity of CTV dye, and adjust the voltage to place PDX-ALL cells that retain the CTV to the right of the dotplot, as seen in Figure 1D.
  i. Once all parameters have been set correctly, use the dashboard to ‘record data’ for each sample.
    i. Export the data as FCS files onto a USB to further analysis through FCS Express 7 Plus Software. Alternative software compatible for similar analysis would include FlowJo.
11. FCS Express Analysis of FACS data.
  a. Open FCS Express 7 Plus to create ‘New Layout’. Select the Insert tab on the workspace window and then select the ‘dot’ option under 2D plots.
    i. Click the cursor on the blank page and open relevant FCS files.
  b. Gate the cells as in step 7.g., to select *intact ALL cells*, and exclude debris, granulated, and the larger MSC cells (Figure 3A).
  c. Display the cells on a new dot plot using FSC-H versus FSC-A parameters. Select ‘Overlays’ at the ‘Gate’ option, and chose *Intact Cells* for display.
    i. Use the polygon gating tool to select *single cells* as indicated in Figure 3B.
  d. On a fresh dot plot display events along FSC-A versus PI-A parameters, and select the ‘gate’ for single cells, as described in 8.c.
    i. Using the Gating tab, select the PI negative population as seen in Figure 3C, these are known as *live cells.*
  e. On a fresh dot plot select events along FSC-A versus CTV-A parameter, and display *live cells* (Figure 3D).
    i. This plot indicates proliferation, where the cells that have retained the full intensity of the CTV labeling will sit on the right of the dot plot and as the cells proliferate, the CTV fluorescence will move more left along the dot plot.
  f. Using the ‘histogram’ option plot cell counts against CTV-A parameters.
    i. Generate histogram ‘Overlays’, and at the gate option, display *Live Cells,* as seen in Figure 3E.
  g. Within the ‘1D plots’ tab, click ‘fit’ and select the ‘proliferation’ plot.
    i. Plot cell counts against CTV-A to show each cell generation.
    ii. Right click on the plot and select ‘statistics’ then ‘population proliferation statistics’ to show the number of cells found in each generation.

### Cell cycle analysis of Hoechst Pyronin Y stained PDX-ALL cells

**Timing: 2 h total**

**Timing: 1 h for step 12**

**Timing: 1 h for step 13**

**Figure 4:**
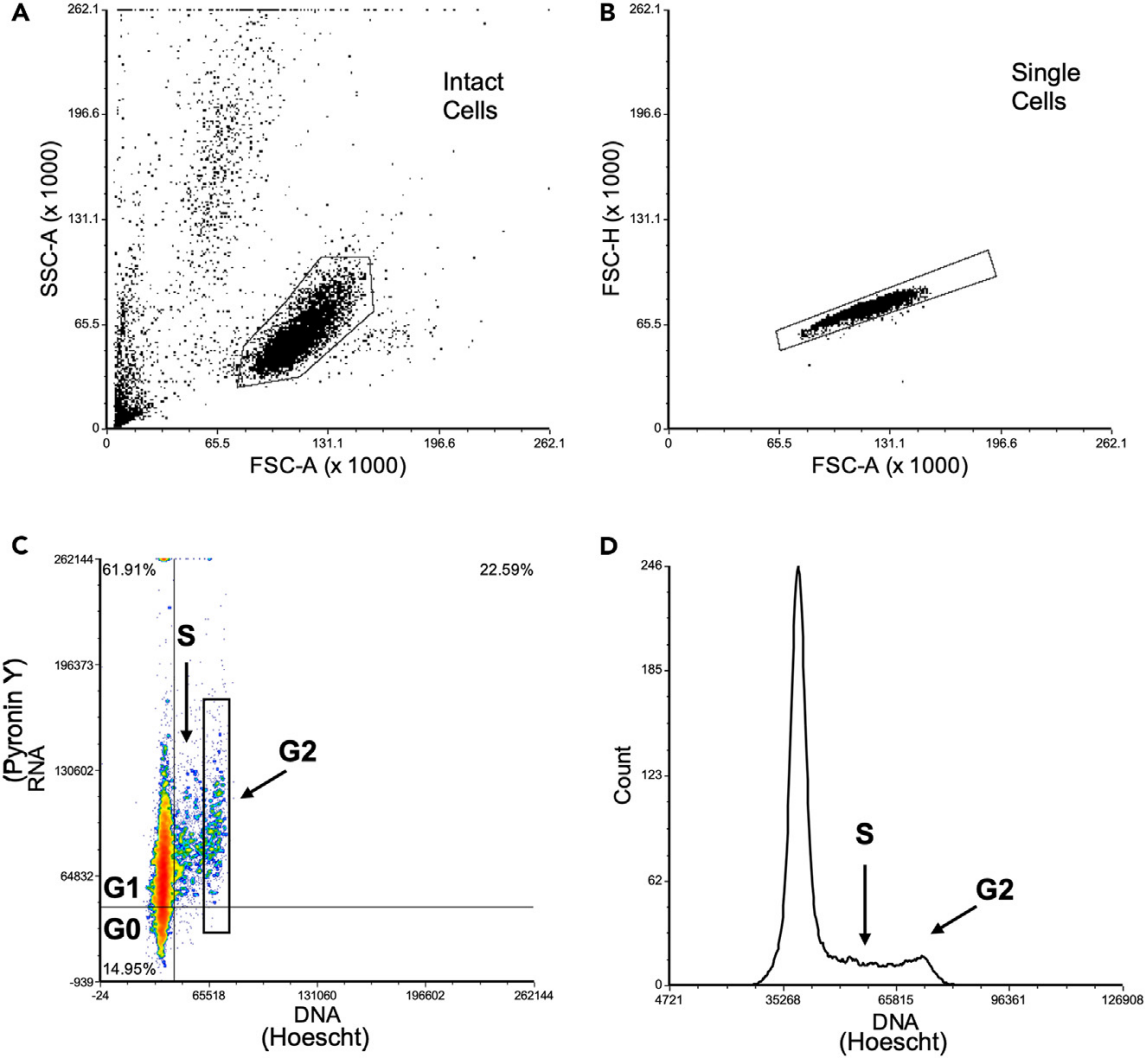
FCS Express analysis of Hoechst Pyronin Y stained PDX-ALL cells (A) SSC-A versus FSC-A to gate intact cells. (B) FSC-H versus FSC-A to gate single cells. (C) Pyronin Y versus Hoechst to show the DNA/RNA content of the cells to distinguish the different stages of the cell cycle and separate G0/G1 cell cycle phases. (D) Cell Cycle analysis of PDX-ALL cells, using Hoechst dye.

This section details the cell cycle analysis of the Hoechst Pyronin Y stained PDX-ALL cells through flow cytometry. This section refers to Figure 4.

12. Flow Cytometry analysis using the Data-Interpolating Variational Analysis (DIVA) software on the FACS Canto II machine.
  a. Set-up the FACS Canto II machine using the standard SOP from BD Biosciences, as detailed in the previously mentioned steps 7 a-c.
  b. In the Cytometer window, click on the Parameters tab. As a default FSC/SSC is in linear scale and fluorescence of dyes is in log scale, all fluorescence parameters were changed to linear scale.
  c. The worksheet window will create dot plots for SSC/FSC and Pyronin Y/Hoechst, for adjusting voltage when acquiring data.
  d. Take the control sample tube and resuspend gently, load the tube onto the SIT, and use the acquisition dashboard to ensure the flow rate is low, SIT flush is selected, number of events is 10,000 and then to ‘acquire data’.
  e. Adjust FSC and SSC voltage to select for the population of intact ALL cells (Figure 4A) and Hoechst indicates levels of DNA, therefore Hoechst needs to be adjusted in view as seen in Figure 4C.
    i. Pyronin Y levels in the presence of Hoechst staining indicate RNA levels.
  f. Once all parameters have been set correctly, use the dashboard to ‘record data’ for each sample.
    i. Export the data as FCS files onto a USB to further analyze through FCS Express 7 Plus Software.
13. FCS Express Analysis of FACS data.
  a. Open the FCS Express 7 Plus and follow Step 8a and 8b to begin analysis, gate *Intact cells* as shown in Figure 4A.
  b. Insert a new dot plot and plot events along SSC-A versus FSC-A parameters.
    i. Select the Gating tab and draw a polygon gate to select the *intact cell* population of cells as identified in step 7.g. (Figure 4A).
  c. Plot events along FSC-H versus FSC-A.
    i. The cursor needs to be double clicked on the dot plot title to display an options menu, using the drop-down bar select ‘Overlays’ and at the ‘Gate’ option select “*Intact Cells”* population.
    ii. Using the polygon gating tool gate in *single cells* for further analysis (Figure 4B).
  d. Use a dot plot along Pyronin Y-A versus Hoechst-A to select and ‘gate’ for *single cells.*
    i. In this graph, Pyronin Y dye represents the RNA content and the Hoechst-A represents Hoechst 33342 staining and therefore DNA content.
    ii. Use a Quad gate as shown in Figure 4C, to distinguish the different stages of the cell cycle by the contents of DNA/RNA within the cells, where Q4 is G0, Q1 is G1 and Q2 is S phase/G2.
      **CRITICAL:** Hoechst stains DNA while Pyronin Y reacts with both DNA and RNA, however in the presence of Hoechst the reaction between Pyronin Y and DNA is blocked and Pyronin Y stains only RNA.
  e. Using the ‘Histogram’ option display *single cells* to show the cell cycle model, select Hoechst for the x-axis as shown in Figure 4D.

## Expected outcomes

Understanding how patient-derived ALL cells behave within their cellular microenvironment, in terms of proliferation and dormancy, is important to reveal key information on treatment resistance. Patient-derived leukemia cells rapidly undergo apoptosis *ex vivo*,^3^ therefore the development of a co-culture system that supports *in vitro* proliferation of PDX-ALL cells is important to facilitate clinically relevant target discovery and treatment development.

**Figure 5:**
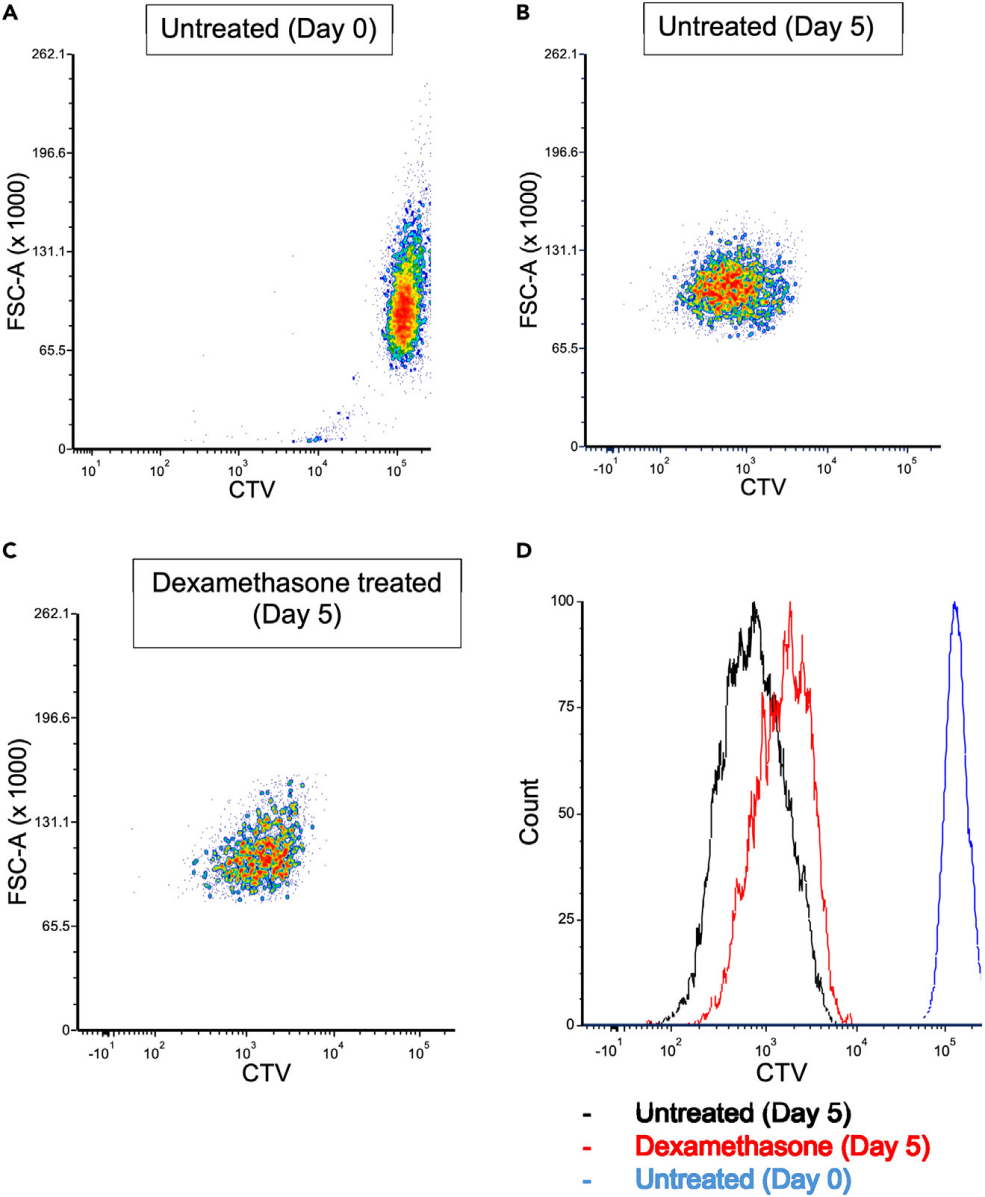
Expected outcomes of CTV labeling in PDX-ALL cells, MS40, after co-culture with MSCs (A) The dot plot displaying the retention of CTV dye in PDX-ALL cells immediately after CTV dye labeling (day 0). (B) The dot plot displaying retention of CTV dye in untreated PDX-ALL cells after 5 days. (C) The dot plot displaying retention of CTV dye in PDX-ALL cells treated with dexamethasone (10 nM) after 5 days. (D) Overlay histogram for untreated day 0 (blue), untreated day 5 (black) and dexamethasone treated day 5 (red) patient-derived cells. Peaks shown further right on the histogram have retained a higher level of CTV dye indicating these cells have cycled slower, compared to peaks more to the left on the histogram which have retained less dye and cycled faster.

**Figure 6:**
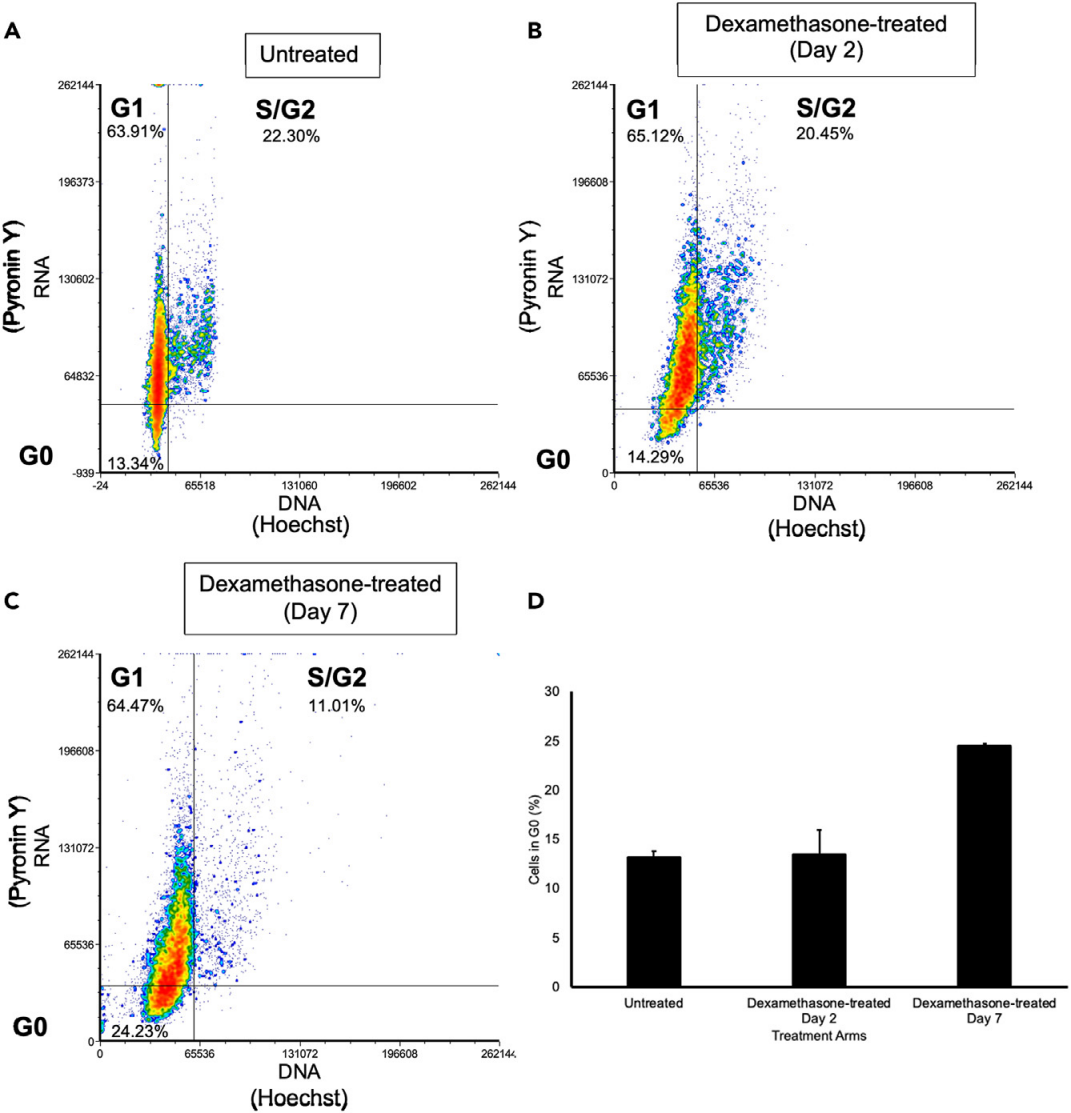
Analysis of Hoechst and Pyronin Y stained PDX-ALL following co-culture with MSC (A) Hoechst Pyronin Y analysis of untreated PDX-ALL cells, L707, indicating which stage of the cell cycle the cells reside in. (B) Hoechst Pyronin Y analysis of 2.5 nM dexamethasone treatment on PDX- ALL cells, L707, after 2 days. (C) Hoechst Pyronin Y analysis of 2.5 nM dexamethasone treatment on PDX- ALL cells, L707, after 7 days. (D) Percentage of PDX-ALL cells in G0 when in the absence of treatment, after 2 days of 2.5 nM dexamethasone treatment and after 7 days of 2.5 nM dexamethasone treatment. *N* = 3.

These protocols enable analysis of cell generational tracking and detection of quiescent PDX-ALL cells harvested following five days of co-culture with MSC (Figures 5 and 6). In summary, this paper describes methodology to generate a co-culture system using PDX-ALL and human MSCs as a key representative of the mesenchymal cellular niche. Furthermore, we describe how to tag PDX-ALL cells with CTV to track slow and fast cycling PDX-ALL in MSC-co-cultures, and stain MSC-primed PDX-ALL cells with Hoechst-33342 / Pyronin Y to detect quiescent niche-primed PDX-ALL. These protocols can be adapted for use in patient-derived leukemia cells representing AML as well as chronic leukemia, and furthermore adapted to include primary leukemia cells that have been directly harvested from clinical samples.

CTV labeling shows the cell generational tracing of PDX-ALL under untreated and dexamethasone-treatment pressure, showing untreated control cells to be actively cycling whilst dexamethasone treatment to reduce cycling of PDX-ALL, resulting in right-hand shift in the CTV curve for dexamethasone-treated-PDX-ALL (Figures 5A–5C).

Hoechst Pyronin Y staining is used to distinguish cell cycle phases in live PDX-ALL cells, specifically to obtain PDX-ALL fractions in G0/quiescent phase. When PDX-ALL are co-cultured with MSC under dexamethasone pressure, MSC-primed PDX-ALL cells show a decrease in S phase. An increase in G0 phase of the cell cycle is observed in MSC-primed PDX-ALL under dexamethasone treatment pressure (Figures 6A–6C). An increase in G0 PDX-ALL continues from day 2 of treatment to day 7 of treatment (Figure 6D).

## Limitations

A key consideration with cell generational tracing experiments, especially those using technically challenging cell types and complex multicellular co-culture models, is selection of optimal *in vitro* labeling dyes.^2^ Emphasis needs to be given to selection of a dye that has low cell toxicity, can label cells brightly with optimal peak resolution whilst ensuring high fidelity dye distribution to daughter cells and demonstrates long-term stability. In this study we use CellTrace Violet as our dye of choice, and furthermore, gate dead cells out of our analysis via PI staining. Furthermore, additional optimization experiments could include conducting longer and/or PDX-ALL co-cultures under improved conditions that might facilitate detection of clearly distinct division peaks. This study uses primary human MSC, and using iPSC-MSC may be more suitable when aiming to standardize such complex muti-step assays. This is because iPSC lines can provide a continuous cell line source, for routine extraction of MSC, via standardized protocols.^1^ Including cell types representing additional cellular constituents of the human bone marrow such as osteoblasts, endothelial and immune cells would furthermore be advantageous, as the bone marrow is diverse and leukemia cell-interaction with different bone marrow cell types mediates cancer biology including treatment response.^1^^,^^6^^,^^7^ Finally, 2D models do not faithfully capture the spatial complexity, including polarity of cell-cell contact and communication.^8^^,^^9^ In this respect, 3D models would show higher biomimicry.^9^

## Troubleshooting

### Problem 1

Limited viability of MSC, MSC do not adhere to flask within 24 h of seeding and/or fail to show one doubling even after five days. This refers to section “PDX-ALL co-culture with MSC”, step 1.

### Potential solution

This could be due to improper Vitronectin coating, where the coating has not been left on for a minimum of 8 h. This could also be due to improper use of Vitronectin-coated flasks, where coated flasks have been left in the incubator and/or at 15–20°C beyond 1 week. FBS used in MSC-media should be routinely batch-tested. bFGF need to be added immediately prior to using MSC-media, bFGF reconstituted MSC-media can be stored at 4°C for a maximum of 1 week.

Furthermore, this could be due to quality of MSC used. We recommend using early passage MSC between passage 2-5. In addition, MSC cultures should not be allowed to reach >80% confluence, that is, individual MSC should not be allowed to clump. MSC can be expanded in tissue culture such that these are frozen in aliquots of early passage MSC. MSC from donors <70 years of age are also preferable where possible. MSC should be regularly test for expression of CD105, CD90, CD73, and for absence of expression of hematopoietic markers such as CD45. Regular functional characterization id advised to ensure MSC retain ability to into osteoblasts, adipocytes and chondrocytes.

### Problem 2

Limited PDX-ALL viability and/or proliferation on MSC. This refers to section “PDX-ALL co-culture with MSC”, step 1.

### Potential solution

This could be due to poor quality of MSC, where MSC do not proliferate adequately, and/or if at some point MSC were beyond 80% confluent and underwent cell-contact-induced senescence. See troubleshooting problem 1. PDX-ALL proliferation is furthermore sample dependent, with some samples requiring seeding densities as high as 2 × 10^6^/mL and some others requiring seeding densities of 0.25 × 10^6^/mL. This generally mirrors time taken to engraft *in vivo*, that is, samples that take longer to engraft *in vivo*, tend to require higher seeding densities *in vitro*. These samples also take longer to double *in vitro*.

### Problem 3

CTV staining does not show any changes from D0 staining. This refers to section “CTV labeling of PDX-ALL cells”, step 4.

### Potential solution

This happens when the PDX-ALL have not sufficiently cycled on the MSC. See troubleshooting problems 1 and 2. Some samples may require longer PDX-ALL-MSC co-culture periods to show proliferation.

### Problem 4

Hoechst staining is suboptimal or absent. This refers to section “Hoechst Pyronin Y staining of PDX-ALL cells”, step 8–9.

### Potential solution

This happens when the dye has gone off. Use a fresh Hoechst 33342 1 mg/mL stock aliquot from −20°C. Do not use any Hoechst stock aliquots that were left at 4°C beyond 1 week.

### Problem 5

Adjusting optimal cell event rate during flow cytometry analysis. This refers to section “proliferation Analysis of CTV labeled PDX-ALL cells” and “cell cycle analysis of Hoechst Pyronin Y stained PDX-ALL cells”, step 10–11 and 12–13.

### Potential solution

Cells were passed through a cell/sample strainer (pluriStrainer) to remove cell clumps and large debris. Samples were then run at “low” sample flow rate and sample flow rate was adjusted to ensure cell event rate was between 300–500 per second.

### Problem 6

Low number of event/s during flow cytometry analysis. This refers to sections “proliferation analysis of CTV labeled PDX-ALL cells” and “cell cycle analysis of Hoechst Pyronin Y stained PDX-ALL cells”, steps 10–11 and 12–13.

### Potential solution

In addition to turning the machine flow rate up, cells were spun down and resuspended at higher densities. Additional measures to improve MSC-PDX-ALL co-cultures have been described in troubleshooting problems 1 and 2.

## Resource availability

### Lead contact

Deepali Pal (deepali.pal@bristol.ac.uk).

### Technical contact

Deepali Pal (deepali.pal@bristol.ac.uk), Jessica Parker (Jess.parker@northumbria.ac.uk), Andrew Filby (Andrew.Filby@newcastle.ac.uk), David McDonald (David.McDonald@newcastle.ac.uk), Carly Knill (Carly.Knill@newcastle.ac.uk).

### Materials availability

Human MSC can be made available upon request, following an MTA.

### Data and code availability

All original data and raw data files have been deposited to Mendeley Data [10.17632/hs9bv4vnsc.1]: Pal, Deepali; Parker, Jessica; Hockney, Sean (2024), “*In vitro* detection of cancer-associated-MSC-primed quiescent and slow cycling cells in leukemia patient-derived co-cultures.”, Mendeley Data, V1, https://doi.org/10.17632/hs9bv4vnsc.1.
